# Chimpanzees employ context-specific behavioral strategies within fission–fusion societies

**DOI:** 10.1007/s10329-024-01165-1

**Published:** 2024-10-19

**Authors:** Jake A. Funkhouser, Stephanie Musgrave, David Morgan, Severin Ndassoba Kialiema, Delon Ngoteni, Sean Brogan, Philip McElmurray, Crickette Sanz

**Affiliations:** 1 Department of Anthropology, Washington University in Saint Louis, One Brookings Drive, Saint Louis, MO 63130 USA; 2 Department of Evolutionary Anthropology, University of Zurich, Winterthurerstrasse 190, 8057 Zurich, Switzerland; 3 Department of Anthropology, University of Miami, 5202 University Drive, Coral Gables, FL 33146 USA; 4 Fisher Center for the Study and Conservation of Apes, Lincoln Park Zoo, 2001 N. Clark Street, Chicago, IL 60614 USA; 5 Wildlife Conservation Society, Congo Program, B.P. 14537, Brazzaville, Republic of Congo

**Keywords:** Niche construction, Multidimensional social network analyses, Behavioral ecology, Behavioral flexibility, Agency

## Abstract

Fission–fusion social systems allow individuals to make flexible choices about where, with whom, and in what contexts to spend their time in response to competing social and ecological pressures. The ability for fission–fusion societies to support individual behavioral strategies that vary across contexts has been suggested, but the potential function of such context-specific social choices remains largely understudied. We adopted the concept of social niche construction to explore possible differences in social complexity at the individual and group level across feeding contexts. Specifically, we examined patterns of co-attendance across two common ecological contexts in wild Central African chimpanzees (*Pan troglodytes troglodytes*) in the Goualougo Triangle, Republic of Congo. From data compiled over 6 years, we used multidimensional social network analysis to study the patterns of co-attendance generated from 436 group scans at *Ficus* and 4527 visits to termite mounds. These two contexts were chosen, because they are both fixed spatial features across the landscape that serve as well-defined points to compare association patterns. We identified context-specific social niche construction in a fission–fusion chimpanzee society that produce different patterns of relationships and social complexity that are consistent in their expression over many years, and offer functional benefits. While enhancing our understanding of chimpanzee behavioral strategies, culture, and conservation, our investigation also indicates that the social niche construction framework aids in elucidating the evolutionary advantages of fission–fusion sociality by accounting for intra- and interindividual variability, cognition, and choice in newfound ways.

**Supplementary Information:**

The online version contains supplementary material available at 10.1007/s10329-024-01165-1.

## Introduction

In most social taxa, individuals employ strategies to maximize the benefits of group living (i.e., mating opportunities, predator defense) while mitigating the costs (e.g., resource competition, disease transmission). Fission–fusion social structures allow individuals to make flexible choices about where, with whom, and in what contexts to spend their time in response to competing social and ecological pressures (Aureli et al. 2008; Chapman et al. 1995). However, conventional models of ecological determinism in primate sociality are limited in their ability to account for the many possibilities of differences in individual behavioral strategies. Such strategies are predicted in societies that display high fission–fusion dynamics where association patterns are flexible and highly variable (Aureli et al. 2008; Strier 2017; Thierry 2008). As an extension of ecological niche theory, the Extended Evolutionary Synthesis accounts for the *social niche* as the set of social conditions required for species-typical social organization and structure as shaped by associations and interactions with conspecifics across multiple, overlapping social networks (Bergmuller and Taborsky 2010; Flack et al. 2006; Laland et al. 2000, 2015; Odling-Smee et al. 1996). Therefore, the collection of social choices by which individuals influence their social environment or select a different set of conspecifics to associate with—i.e., *social niche construction*—sets forth a new series of hypotheses and predictions that are applicable to the study of fission–fusion social systems (Kaiser et al. 2024; Saltz et al. 2016; Trappes 2021; Trappes et al. 2022). Social niche construction is a promising line of inquiry that will generate novel insights into the evolutionary functions and correlates of fission–fusion, beyond the understandings possible under ecological determinism.

**Fig. 1:**
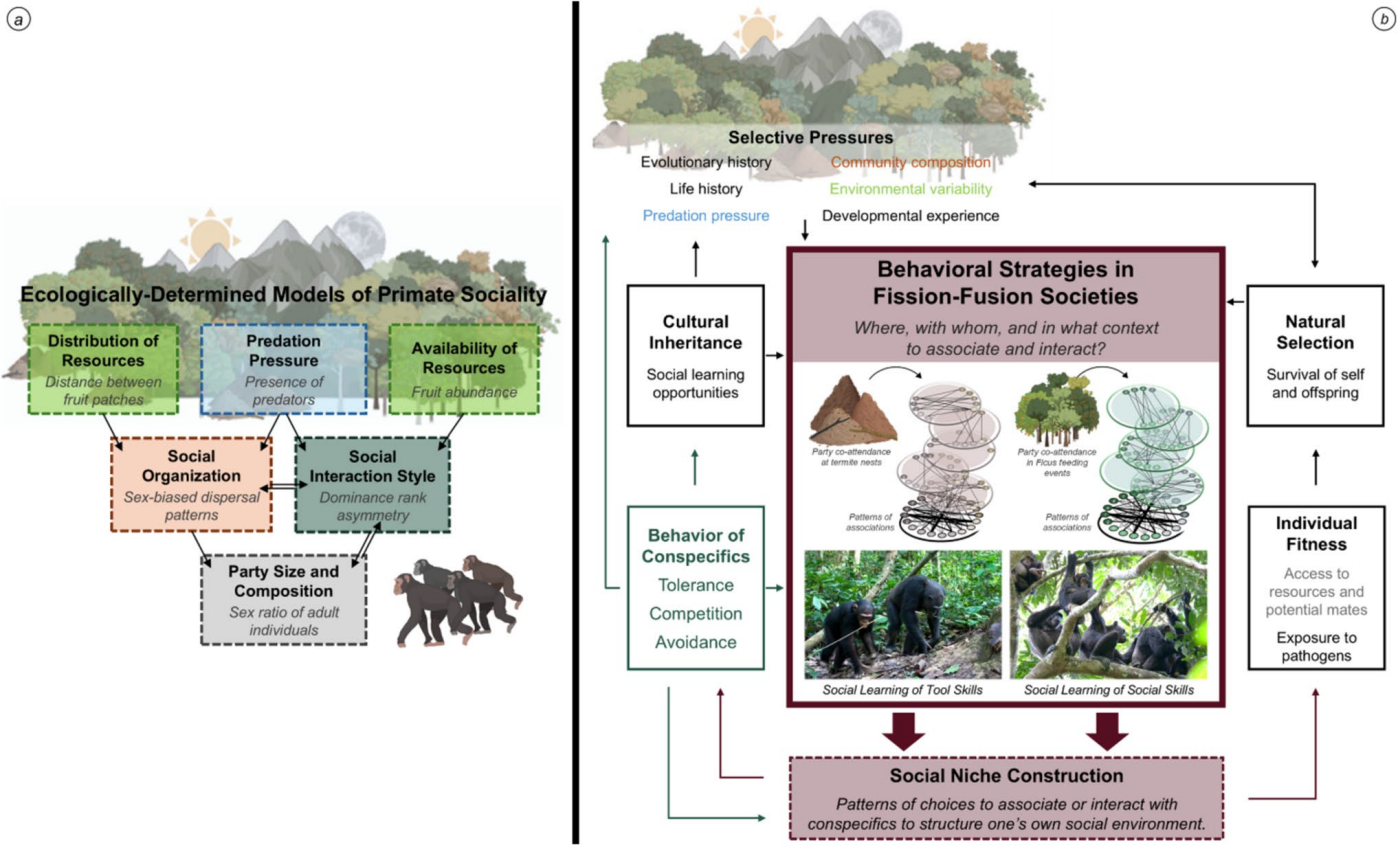
Predictive factors of (**a**) ecologically determined models of primate sociality compared to (**b**) individual variation in behavioral strategies and social niche construction within fission–fusion societies as positioned within the extended evolutionary framework

Social niche construction offers a complementary perspective to ecologically determined models of primate sociality for considering the functional consequences of living in fission–fusion social systems. In this type of social system, individuals make active choices to associate with specific conspecifics in selected contexts or localities within their home range, often at a vast distance from other sets of conspecifics. Under the assumptions of the social niche construction framework, interindividual differences in social choices regarding with whom to associate or interact need not be for entirely social reasons, independent from foraging or other decisions, nor must they coincide with sophisticated cognition for the resulting social niche to influence one’s fitness. Importantly, the social niche construction framework offers a unique assumption in studying fission–fusion social systems, that individuals make choices. From an individual’s choices, their social niche is constructed, thereby influencing their fitness via an interaction with the bidirectional forces that are shared with ecological, cognitive, and other selective pressures (for a complete review, see Kaiser et al. 2024; Trappes 2021; Trappes et al. 2022). In animal societies with cohesive social structures and inflexible or highly consistent social environments, where social niche construction has been predominantly studied, individuals exert choice and agency via patterns of social interactions (e.g., primate conflict interventions: Flack et al. 2006; grooming: Mielke et al. 2021). However, social niche construction has not yet been extended to fission–fusion societies, where individuals are afforded an additional layer of choice in constructing their social environment via patterns of party co-attendance (e.g., associations: Boesch and Boesch-Achermann 2000; McCarthy et al. 2019). The use of social niche construction to study the behavioral ecology of animals living in fission–fusion societies will produce novel insights into individual behavioral strategies (i.e., intraspecific and interindividual variation) that are often occluded by conventional socioecological models (Bergmuller and Taborsky 2010; Clutton-Brock and Janson 2012; Strier 2017; Thierry 2008; also see Fig. 1). The ability to flexibly construct social niches and enact functional behavioral strategies are not concepts currently considered in the comparative investigations of behavioral ecology via the study of standard socioecological variables (e.g., fruit availability, predation pressure, and mating opportunities), although such flexibility forms the foundation of fission–fusion societies.

It has been proposed that the fission–fusion social systems of chimpanzees are adaptive in enabling females and their young offspring to avoid intragroup aggression by varying group cohesion in response to food scarcity while foraging together for plentiful resources, constructing or strengthening a few strong relationships, and cooperating in the avoidance of predators (Boesch and Boesch-Achermann 2000). Meanwhile, this type of social system is hypothesized to enable chimpanzee males to remain in their natal groups and form strong alliances with relatives to defend positions of power and oppose intergroup individuals (Pusey 1980; Wrangham 1980). Additionally, the flexibility of a fission–fusion social system allows males to selectively form and navigate consortships with preferred potential mates (Lehmann and Boesch 2004; Matthews et al. 2021). Although some external and demographic factors predict interspecific variation in fission–fusion party size and sex ratios (Itoh and Nishida 2007; Koomen and Herrmann 2018; Koops et al. 2024; Lehmann and Boesch 2004; Lehmann et al. 2007; Newton-Fisher et al. 2000; Wittiger and Boesch 2013), very little is understood about how differences in individual behavioral strategies shape each chimpanzee’s social environment and produce the variability that is predicted to be inherent to fission–fusion societies. A specific, detailed understanding of the function of fission–fusion social variability in chimpanzees will likely continue to be elusive without new conceptual frameworks. One such framework is social niche construction.

**Fig. 2:**
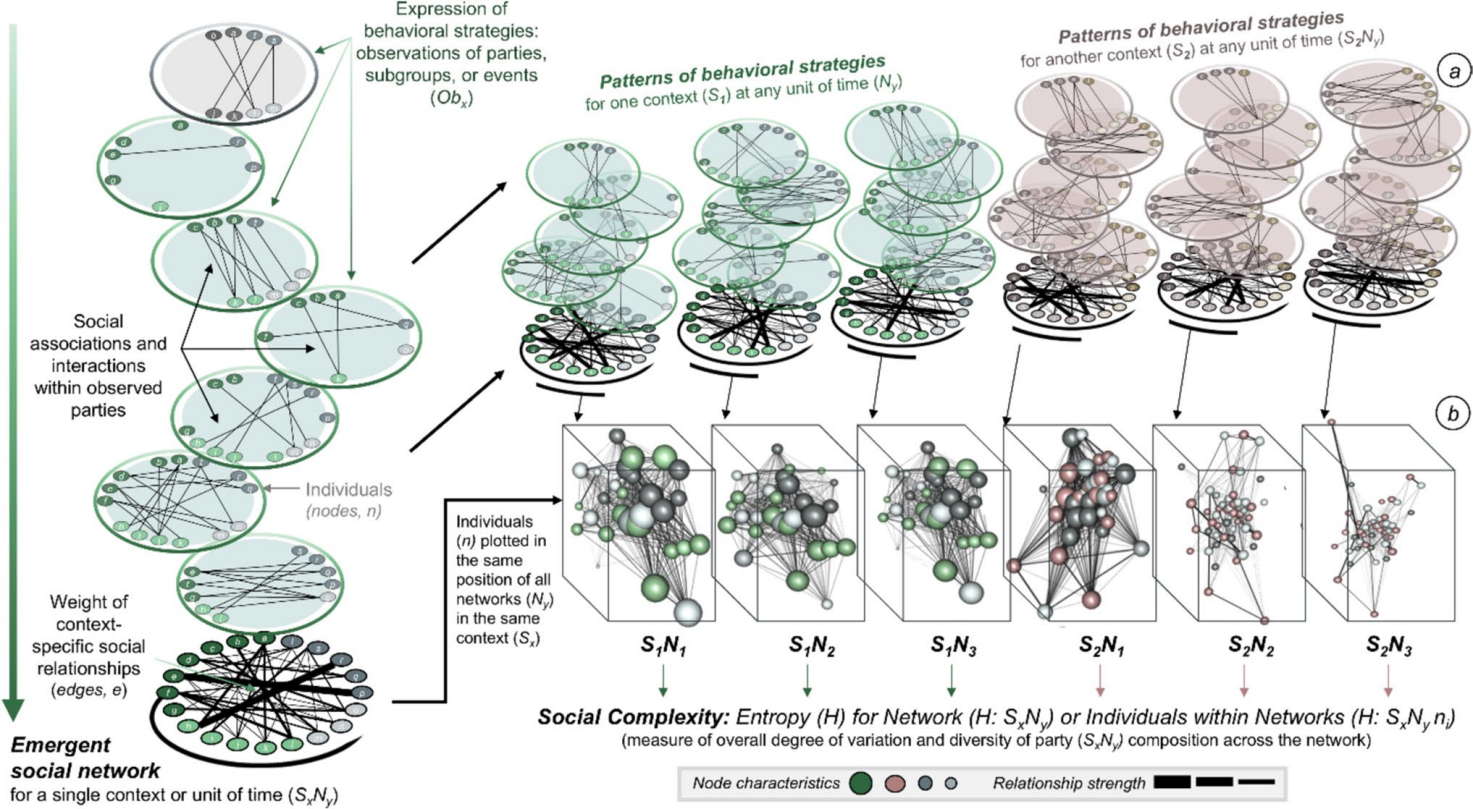
A framework for the study of social niche construction. Social niche construction is scaffolded from (**a**) patterns of repeated associations by individuals (nodes, *n*) across many observations (*Ob*_*x*_) into aggregate weighted social relationships (edges*, e*) within the emergent social network (e.g., Σ*Ob*_*x*_) for each network (*N*_*y*_) across different contexts (*S*_*x*_). Visually, individual and relationship characteristics can be represented as differences in size (e.g., network centrality or importance), color (e.g., node: individual age and sex; edge: relationships strength), or position (e.g., comparable circle layouts, integrated nodes in the center, isolate nodes in the periphery). In constructing their social niches, chimpanzees make flexible social choices that change the emergent patterns of relationships and structures of context-specific networks. The emergent social networks (*S*_*x*_*N*_*y*_) can then be represented as (**b**) layers of a multidimensional social network (e.g., *S*_*x*_*N*_*1,2,3…n*_) that allow for individual network position, patterns of relationships, social structure, and complexity to be analyzed in biologically meaningful units. Drawn with some reference to: Barrett et al. 2012; Hinde 1976

Individual behavioral strategies can be measured by context-dependent patterns of social associations and the resulting relationships. Adult chimpanzees make choices to alter their association with conspecifics in parties of various sizes and compositions across different contexts (e.g., Chapman et al. 1995; Fox et al. 2024; Itoh and Nishida 2007). The ecology of these different contexts can either inform an individual’s choice to associate with others or coincide in non-mutually exclusive ways with the other motivations from which individuals make their choices (e.g., who is there). Such choices, regardless of their motivation, therefore produce variable patterns in where, when, how, and with whom individuals learn and socially interact (Aureli et al. 2008; Pascual-Garrido 2019). The flexible expression of individual behavioral strategies in response to local social or environmental pressures confers numerous evolutionary advantages (Kaiser et al. 2024; Lipatov et al. 2011; Saltz et al. 2016; Trappes et al. 2022). For example, the dynamic maintenance of chimpanzee social relationships could expedite the transmission of cultural behaviors in social and tool using contexts (Fogarty and Creanza 2017; Laland et al. 2001; Sterelny 2007). Therefore, by making choices about where, with whom, and in what context to spend their time, chimpanzees can construct social niches (also see Fig. 2). Differences in the repeatability or variability of party compositions across contexts can, thereby, produce individual-level variation in social complexity (i.e., variation in the diversity of social partners within parties of variable sizes: Ramos-Fernandez et al. 2018). Variation in social complexity across contexts could also confer context-specific benefits. For example, higher social complexity as a result of associating with a variable array and a diverse number of conspecifics could increase one’s reproductive success or expedite one’s acquisition of cultural information. Context-specific behavioral strategies expressed via party co-attendance associations that result in patterns of social relationships can be operationalized via social niche construction to better understand the outcome of flexible fission–fusion social systems. The way that chimpanzee social associations are flexible in their rate, frequency, duration, and diversity allows for interindividual variation in the construction of their social environment.

Flexibility in social niche construction generates interindividual differences in choosing particular contexts and social partners more frequently than others. Such flexibility likely promotes *social niche specialization*, whereby individuals express context-specific behavioral repertoires, skills, knowledge, and/or competencies that allow them to be uniquely successful in specific contexts over many years in a way that might influence their fitness (Bergmuller and Taborsky 2010; Kaiser et al. 2024; e.g., conflict interventions: Flack et al. 2006; von Rohr et al. 2012; boundary patrols: Massaro et al. 2022; coordinated hunting: Boesch 2002; Gilby et al. 2015). At the level of individual chimpanzees, variation in the flexible construction of social niches can provide access to critical benefits in specific contexts. For example, the successful integration of immigrant females depends on their ability to outcompete conspecifics to establish mating opportunities and alliances/coalitions or other strong relationships (Kahlenberg et al. 2008). Additionally, the documented variation in chimpanzee cultural traditions necessitates social learning throughout each individual’s lifespan (Boesch et al. 2020; Whiten and Boesch 2001; Whiten and van de Waal 2018). Immature apes attain culturally specific tool use and social cultural variants via social learning. Even as adults, chimpanzees continue to learn about critical cultural information that relates to social, tool use, and foraging contexts (Biro et al. 2003; Carvalho and McGrew 2010; Luncz and Boesch 2014; Luncz et al. 2012, 2015; Nakamura and Uehara 2004). These benefits are only accessible when individuals make choices to associate with selected collections of conspecifics in a selected context.

Variation in goal-oriented social choices and the construction of social niches would predict that chimpanzees can optimize access to socialization, mating, learning, and acquiring food in variable party sizes and composition across context-specific settings. One context that consistently brings together large chimpanzee parties of all ages and both sexes is *Ficus* feeding events, as their production is asynchronous across the forest. In this context, chimpanzees tend to associate with a more diverse array of individuals and in larger parties than is common elsewhere. Thus, this context offers opportunities to transmit and acquire important social skills and group-specific social customs (e.g., interaction styles: Boesch and Boesch-Achermann 2000; van Leeuwen et al. 2018; Whiten et al. 2005). Another context that represents opportunities for social learning of local cultural variants is tool using sites, such as termite nests, where chimpanzees may choose to associate in small parties to observe and practice tool material selection, modification, and sequence of use (e.g., tool sets in the Goualougo Triangle: Sanz and Morgan 2013). Such selective parties provide opportunities for chimpanzees to associate with other tool users from whom important tool skills and local behavioral variants can be transmitted by a diverse array of proficient models and acquired by novices (Coussi-Korbel and Fragaszy 1995; Sanz and Morgan 2011; van Schaik and Pradhan 2003). Herein, we examine the possibility that individual chimpanzees employ context-specific patterns of individual behavioral strategies across different foraging contexts that result in variation of social complexity.

In this investigation, we tested for individual variation in social niche construction across *Ficus* feeding and termite gathering contexts using multidimensional social network methods of fission–fusion dynamics in wild chimpanzees in the Goualougo Triangle, Republic of Congo (*Pan troglodytes troglodytes*). We hypothesized that intraindividual variation in social niche construction produces different patterns of social relationships across contexts. First, we predicted that the different patterns of social relationships across contexts would be temporally consistent (i.e., stable across time) thus, potentially, influencing one’s fitness. Second, we predicted that different patterns of social relationships across contexts would result in different levels of social complexity, specifically higher complexity in the tool assisted termite-gathering context. Finally, we predicted that different patterns of social relationships across contexts would likely produce context-specific functional advantages, namely via greater information transmission efficiency in the tool assisted termite-gathering context.

This population of chimpanzees is an excellent system to examine social niche construction as the associations of habituated individuals in fission–fusion parties have been longitudinally monitored for many years and across contexts, including during visits to *Ficus* and termite nests. Chimpanzees in the Goualougo Triangle use sets of tools in multiple foraging contexts including termite gathering (Sanz et al. 2009, 2004, 2010; Sanz and Morgan 2009). These chimpanzees flexibly plan their tool use (Musgrave et al. 2023), their tool-use behaviors are socially transmitted to others (Musgrave et al. 2016), and the greater tolerance exhibited by models to learners likely facilitates enhanced social transmission compared to other populations (Musgrave et al. 2020). When chimpanzees in the Goualougo Triangle arrive at termite nests, they use a rigid, woody tool to first puncture into subterranean nests or they use their fingers or a woody twig to first perforate into the nest exterior at above-ground nests (Sanz and Morgan 2011). Then, they select a particular species of flexible herbaceous vegetation to manufacture brush-tipped termite-fishing probes (Marantaceae plants: Sanz et al. 2009). Therefore, research on the flexible construction of social niches in this population is well suited to providing novel insights into the social processes that promote complex tool cultures. An additional aim of this research is to relate our findings to hominin evolution (e.g., how social niche construction expedites the accumulation of cultural information) while also informing the conservation and wellbeing of chimpanzees around the world (e.g., the conservation of cultural variants and promotion of species-typical social behavior).

## Method

We examined patterns of 41 adult (*n* = 27 females, 14 males) and 22 immature (*n* = 17 females, 5 males) wild chimpanzee social relationships across 6 years (January 2014–December 2019) in different contexts within the Goualougo Triangle, Republic of Congo. All individuals included in the analyses were present in the community for at least 3 years. To record party compositions in different contexts, we collected direct group scan observations every 20 min at 79 unique fig trees (*Ficus sp*.) across the community’s home range and scored high-definition 60-s video clips from motion-activated camera traps deployed at 33 termite nests (*Macrotermes* sp.). Across contexts, we defined parties as individuals who associate within the same context (i.e., at the same place) at the same time. To operationalize a metric of co-attendance that would be comparable across foraging contexts, we used validated measures to record all individuals within a 50 m search area at a *Ficus* during group scans (Boesch and Boesch-Achermann 2000; Lehmann and Boesch 2009; Mielke et al. 2017; Murray et al. 2016; Ramos-Fernandez et al. 2018; Reynolds 2005; Samuni et al. 2020; Wilson et al. 2001; Wrangham et al. 1992) and all individuals observed at the same termite nest within 15 min of one another in camera trap footage (McCarthy et al. 2019, 2018). Our combination of data collection methods allowed for precise party composition records of the same individuals to be collected across the *Ficus* feeding context as well as termite nest sites with high resolution. These two types of resources occur at fixed locations across the Ndoki landscape. Although they are relatively rare compared to other types of resources, both are available to all community members and were assessed at multiple points across space and time. Further, neither resource was entirely exploited during any single visit, as indicated by available opportunities for foraging on open branches or termite-fishing tunnels.

### Social network construction and analysis

From the party co-attendance observations in *Ficus* and termite gathering contexts, we constructed an aggregate 2014–2019 social network as well as a network for each calendar year of data collection. We constructed weighted symmetric simple ratio association index matrixes for each network in SOCPROG (Whitehead 2009). The simple ratio calculation for dyadic association indexes is most appropriate when defining a single association as co-attendance or presence in the same sampling period (i.e., party: Ginsberg and Young 1992; Whitehead 2008a). The equation directly below provides the calculation for a simple ratio association index (*SRI*), where *x* is the number of sampling periods where individuals *a* and *b* were observed together, and *y* is the number of sampling periods where *a* and *b* were observed elsewhere.

$${\text{Simple Ratio Association Index}}:{\text{ SRI}} = \frac{X}{{X + { }Y_{a} + { }Y_{b} }}$$

### Tests for context-dependent patterns of relationships

We hypothesized that intraindividual variation in social niche construction produces different patterns of social relationships across contexts. To test this hypothesis, we tested the 2014–2019 aggregate networks in both contexts against the null hypothesis that individual chimpanzees were equally as likely to associate with all other individuals (i.e., observed mean and standard deviation association indexes that are greater than randomly generated values, or chance: Whitehead et al., 2005; Whitehead et al. 2008a). Like other matrix permutation tests, these analyses in SOCPROG swap observed matrix values randomly across the axis at each iteration (Bejder et al. 1998; Farine 2017). In most cases, 1000 iterations are sufficient permutation tests of network and individual node-specific metrics (Whitehead 2008a, 2009).

To evaluate interindividual differences in social niche specialization and overall network characteristics, we computed several commonly used node (individual) and network metrics using degree, edge_betweenness, strength, eigen_centrality, and transitivity functions with iGraph 1.3.0 in R 4.1.3 (Csardi and Nepusz 2006; R Core Team, 2021).

Degree centrality counts the number of conspecifics that the node shares a direct relationship with at any strength. Degree centrality is helpful in understanding the foundational number of possible routes of transmission in a network (Croft et al. 2016; Sosa et al. 2020; Wey et al. 2008). Betweenness centrality counts the number of direct two-step transmission paths that run through an individual. Betweenness centrality can estimate the efficiency or speed of transmission in a network (Croft et al. 2016; Sosa et al. 2020; Wey et al. 2008). Strength centrality sums the weight (association index) of all direct relationships with conspecifics. Strength centrality helps evaluate network structure, because it is standardized by simple ratio association indexes (Croft et al. 2016; Sosa et al. 2020; Wey et al. 2008).

*Eigenvector centrality* is a measure of an individual’s structural importance in a group based on network position, the number, and weight of direct relationships, as well as the number and weight of indirect relationships (at one degree of separation). Eigenvector centrality is particularly ideal for comparisons across individuals and networks as it is reported on a standardized 0–1 scale and is not affected by small differences in sampling (Bonacich 2007; Farine and Whitehead 2015; Newman 2004; Pasquaretta et al. 2014). The equation directly below provides the calculation for eigenvector centrality where *x* centrality of individual *i* is proportional to the sum of the centralities of all the relationships that are connected to it, *a* is the association matrix, $\uplambda$ is the largest eigenvalue of *a*, and *n* is the number of other *j* nodes

$${\text{Eigenvector centrality}}:{ }x_{i} = { }\lambda x,\lambda x_{i} = \mathop \sum \limits_{j = 1}^{n} a_{ij} x_{ij} , i = 1, \ldots , n.$$

Clustering coefficient or transitivity is a measure of node isolation or disintegration that estimates the degree to which the possible transmission pathways to and from a node are restricted to a discrete subgraph with less than the expected number of connections (Barrat et al. 2004; Croft et al. 2016; Newman 2006; Pasquaretta et al. 2014). Transitivity can help estimate the speed of transmission between small groups of well-connected network members as well as detect modularity across the network (significant decay in connectedness). The equation directly below provides the calculation for clustering coefficient where *s*_*i*_ is the strength of node *i*, *a*_*ij*_ are the elements of the association matrix, *k*_*i*_ is the degree of node *i*, and *w*_*ij*_ is the weight of the relationship between node *i* and *j*

$${\text{Clustering coefficient}}:{ }C = \frac{1}{{s_{i} \left( {k_{i} - 1} \right)}}{ }\sum j,{ }h{ }\frac{{w_{ij} + { }w_{ih} }}{2}{ }a_{ij} a_{ih} a_{jh} .$$

*Network density* is a measure of the connectedness of all members of an entire network. That is, the probability that a relationship was observed between any dyad or the number of observed relationships divided by the total number of possible relationships. Network density is ideal for comparing the structure of both paired and independent networks, because it can be interpreted to indicate the approximate relative efficiency of possible transmission. For example, if a difference of 0.03 in density was observed between network *X* and network *Y* (assuming equal rate and temporal characteristics of associations), this result would indicate 3% faster transmission across 3% more individuals in network *X* compared to network *Y*. Additionally, network density is not affected by networks of different sizes nor is it biased by differences in sampling (Borgatti et al. 2013; Sosa et al. 2020; Wey et al. 2008). The equation directly below provides the calculation for weighted symmetric network density where *N*_*o*_ is the number of observed relationships and *n* is the number of network nodes

$${\text{Density}}:{ }D = \frac{{N_{o} }}{{{{n\left( {n - 1} \right)} \mathord{\left/ {\vphantom {{n\left( {n - 1} \right)} 2}} \right. \kern-0pt} 2}}}$$

We tested for differences in the network structure (density) of the Ficus and termite gathering aggregate networks using bootstrapped permutation tests where the observed densities are tested against the distribution of 1000 possible probabilities that a relationship exists between two randomly chosen nodes (Lusseau 2003; Manno 2008; Shizuka and Johnson 2020); this analysis is built into UCINET (Borgatti et al. 2013).

We also tested for differences in the distribution of node-specific degree, betweenness, strength, eigenvector centrality, transitivity, and social complexity across the aggregate Ficus and termite gathering context using bootstrapped paired samples *t* tests with MKinfer (“boot.t.test()” with 1000 permutations) in R 4.1.3 (Kohl, 2022; R Core Team, 2021).

### Test for temporal consistency of context-dependent patterns of relationships

We predicted that context-dependent patterns of relationships would be consistent across time, thus, potentially, influencing one’s fitness. Using the simple ratio association index matrixes that we calculated for each year of data collection in both contexts, we constructed a multidimensional social network with data from each year stored as individual layers (MuxViz: De Domenico et al. 2014). We then employed the standard bootstrapped Pearson’s correlation procedure across layers within each context to test for consistency and stability in social network structure across time as would be indicated by significant edge overlap (Riesch et al. 2012; Smith-Aguilar et al. 2019).

### Test for context-specific metrics of social complexity

We predicted context-specific social niche construction would produce different levels of social complexity across contexts. Shannon’s entropy has been validated as a useful general metric of the overall degree of variation and diversity (i.e., uncertainty) of subunit (i.e., party) compositions that make up a social network both at the group and individual level. Thus, it is an ideal metric for assessing both interindividual variation in individual behavioral strategies across contexts as well as the emergent results of these different behavioral strategies at the group level. Further, in validation tests, the algorithm was found to be resistant to influence from differences in sample size and party size, making it useful in the social complexity across contexts (Ramos-Fernandez et al. 2018). This algorithm outputs both observed (H) and bootstrapped (H_b_) values of group and individual-level entropy (*H*_*i*_*, H*_*bi*_), where H represents a comparative measure that ranges from 0 probability of uncertainty one would have about the composition of a party chosen at random. This algorithm also provides JS distance scores (i.e., the difference between H and H_b_), which helps to infer whether the observed entropy is significantly larger or smaller than the bootstrapped maximum entropy that would be expected in a dataset of the same size and subunit size distribution if all compositions were equally likely. We calculated Shannon’s entropy (H) values using the source code and analysis protocol with minor modifications to include individuals who were only observed once in each context and parties of only one individual (Ramos-Fernandez et al. 2018). We report observed group and individual-level entropy scores (*H, H*_*i*_) and *JS* distances as these are likely the best available metrics for understanding variation in social complexity within fission–fusion social systems

$$p_{\left[ a \right]} = \frac{{{\text{number of observed subsets}} = \left[ a \right]}}{{\text{total number of subsets}}},$$

and,

$${\text{Entropy}}:{ }H = - \sum p_{\left[ a \right]} { }log_{2} p_{\left[ a \right]} ,$$

To calculate social complexity at the individual level (*H*_*i*_), the same formula as above is applied but only to subgroups within which the selected individual (*i*) was observed and by considering unique compositions of parties in terms of the remaining *n*–1 individuals. Equations for bootstrapped entropy (*H*_*b*_*, H*_*bi*_) and JS distances, along with all other details of these calculations, are reported with their source code and analysis (Ramos-Fernandez et al. 2018).

All research reported in this manuscript complied with the protocols approved by the Washington University in St. Louis Institutional Animal Care and Use Committee and the legal requirements of the Republic of Congo. This research also adhered to the International Primatological Society’s code of best practices for field primatology.

## Results

We constructed the *Ficus* network from chimpanzee party co-attendance in 436 group scan observations: mean party size ± SD = 5.92 ± 4.70 individuals. Additionally, we constructed the termite gathering network from chimpanzee party co-attendance in 30,649 camera trap clips (n = 4527 visits composed of 5 ± 7.5 clips): mean party size ± SD = 1.76 ± 1.21 individuals.

We confirmed that the observed patterns of chimpanzee associations were different from networks that would be expected by randomly permuted datasets in the *Ficus* (2014–2019 observed mean [0.08] and standard deviation [0.13] was greater than randomly generated mean [0.07] and standard deviation [0.07]; *P* < 0.001; 1000 permutations; range 0.0–1.0) and termite gathering contexts (2014–2019 observed mean [0.013] and standard deviation [0.07] was greater than randomly generated mean [0.011] and standard deviation [0.04]; *P* = 0.001; 1000 permutations; range 0.0–1.0: Bejder et al. 1998; Whitehead 2008b).

**Fig. 3:**
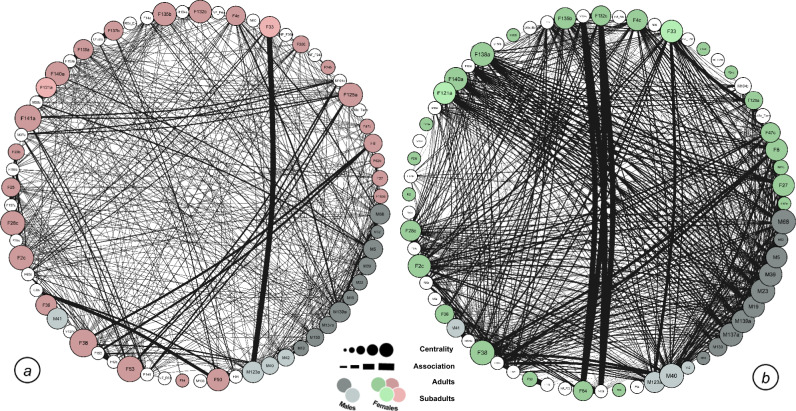
Expression of context-specific behavioral strategies in patterns of relationships in (**a**) termite gathering and (**b**) *Ficus* feeding contexts. Node placement for each individual is held consistent across both network graphs to illustrate changes in network strength centrality (node, circle size) and patterns of relationships (edge, line thickness). Each chimpanzee’s node size is scaled to network centrality and relationship strength is scaled to line thickness. Adult male nodes are gray, adult female nodes are colored, and immature nodes are white. Node color darkens with age. The structure of these networks across contexts is significantly different

We also confirmed that the structure of the *Ficus* and termite gathering networks were significantly different from one another (*difference in density* = 0.03; bootstrapped paired T test with 1000 permutations = 6.04; 95% CI = 0.021–0.042; *P* value = 0.002). Figure 3 illustrates the different patterns of relationships (lines between individual nodes) in termite gathering (Fig. 3a) and Ficus (Fig. 3b) network structures.

Temporal consistency of network structure and relationship patterns were also evident in the multidimensional social networks that we constructed using data from each year of data collection as individual network layers. There was considerable overlap between the structure of all network layers in *Ficus* (mean edge overlap = 0.37) and termite gathering contexts (mean edge overlap = 0.39) (Liu et al. 2018). Detailed interlayer correlation results for each pairwise comparison in both contexts are reported in Supplementary Information Table S1.

At the individual level, an analysis of the distribution of average party sizes that each chimpanzee was observed in indicates a presence of social heterogeneity across all individuals in *Ficus* (2014–2019 observed mean number of partners = 3.36, expected at random = 2.50; P = 0.001) and termite gathering contexts (2014–2019 observed mean number of partners = 0.85, expected at random = 0.78; P = 0.003: Whitehead 2008b). Within these analyses, there was also evidence of both homophily (individuals who were consistently observed in parties with *more* conspecifics than would be expected by chance: *Ficus, n* = 7; termite gathering, *n* = 3) and heterophily (individuals who were consistently observed in parties with *fewer* conspecifics than would be expected by chance: *Ficus, n* = 8; termite gathering, *n* = 1), thereby serving as another indicator of considerable interindividual variation within contexts and the potential for evidence of social niche specialization by certain individuals.

The calculated network centrality of individual chimpanzees also differed significantly across the *Ficus* and termite gathering contexts (bootstrapped paired t test with 1000 permutations: *degree centrality*: *T* (64) = 3.54, CI = 2.55–8.95, *P* = 0.003; *betweenness*: *T* (64) = 15.11, CI = 14.10–32.18, *P* < 0.001; *strength centrality*: *T* (64) = 7.43, CI = 1.47–2.54, *P* < 0.001; *eigenvector centrality*: *T* (64) = 4.36, CI = 0.13–0.35, *P* < 0.001), except transitivity (*T* (64) = 0.90, CI = -0.07–0.19, *P* = 0.38).

Furthermore, the social complexity displayed by population-level dynamics as well as the social complexity displayed at the individual-level (i.e., experienced by individuals) differed across the *Ficus* (*H* = 8.65) and termite gathering contexts (*H* = 9.29): bootstrapped paired t test with 1000 permutations, individual-level social complexity (*H*_*i*_), *T* (64) = 4.10, CI = 0.61–1.72, *P* = 0.001; mean of intraindividual differences across contexts = 1.16.

## Discussion

We aimed to determine if chimpanzees in the Goualougo Triangle employ context-specific individual behavioral strategies within their fission–fusion society that support predictions of social niche construction. We found temporally consistent, context-dependent patterns of social niche construction by the same individuals across two different foraging contexts: feeding on figs (*Ficus*) and termite gathering. From data compiled over 6 years, we used multidimensional social network analysis to examine longitudinal records of where and with whom these chimpanzees chose to spend their time when visiting *Ficus* or termite nest foraging contexts. Our results indicate that the flexible construction of social niches in fission–fusion social systems produces temporally consistent and functionally beneficial social niche specializations in chimpanzee societies. These results offer new insights into the function of fission–fusion sociality, pointing toward social niche construction increasing context- or skill-specific benefits. The possibility of ape specialists functioning to expedite the context- or skill-specific learning of conspecifics has broad implications for models of the evolutionary emergence of cumulative culture in hominin evolution (Muthukrishna and Henrich 2016; Smolla and Akcay 2019). The types of evolutionary implications offered by this investigation are only possible through our use of the social niche construction framework to study fission–fusion sociality.

Compared to other social structures, fission–fusion social systems grant individuals greater agency to make flexible decisions about where, with whom, and in what contexts they spend their time (Aureli et al. 2008). The ability to make these kinds of choices is adaptive in allowing individuals to experience the benefits of living in large groups while mitigating the costs of intragroup competition for access to resources when they are difficult to find (Malone et al. 2012). Our evidence of context-specific social niche construction promotes the idea that individuals within fission–fusion social systems are free to employ different individual behavioral strategies by selecting to spend time with specific parties of conspecifics. These kinds of behavioral strategies likely produce functional, beneficial outcomes. For example, choosing to engage in social contexts (e.g., in *Ficus* feeding contexts) likely permits individuals opportunities to construct and maintain a vast network of social relationships that incurs benefits, such as attaining greater social power, access to preferred mates, or socialization opportunities for their offspring. Meanwhile, specializing in tool using contexts (e.g., in termite gathering parties) likely incurs a separate set of benefits such as acquiring local tool-use cultural variants within the company of a few highly tolerant individuals, gaining access to termites as a nutritional resource, maintaining discrete consortships with selected mates, and providing offspring with the opportunity to learn complex tool tasks from expert models. Of prominent interest moving forward will be to explore how cognitive abilities (e.g., planning, theory of mind: Krupenye and Call 2019) interact with social niche construction processes and their fitness-related outcomes. Although our study was conducted over only 6 years, the temporal consistency of social niche construction in this chimpanzee population supports the idea that evolutionary mechanisms also influence these patterns of adult social behavior given their repeated expression over time and possible links to reproductive success. Future lines of research that draw from a greater array of contexts to compare varying sets of evolutionarily advantageous skills produced by goal-oriented choices that correspond to discernable motivations will aid in disentangling the bidirectional relationships across ecological, social, and cultural factors that may influence social niche construction processes. We also acknowledge that future research is necessary to inform a comprehensive account of the correlates and function of fission–fusion, including alternative explanations which may include that the temporal availability and distribution of food resources could compose most of an individual’s motivations to make the choices from which their social niche is constructed. Therefore, future tests are necessary to discern to what degree aspects of ecology influence the patterns of individual choices that we describe here to construct social niches and produce the observed functions of fission–fusion sociality. Nonetheless, the results of our current investigation help to better understand the costs and benefits of fission–fusion social systems along with the types of strategies individuals might employ in such a system that affords greater levels of choice and agency. Our results also aid in elucidating new aspects of the selective pressures that hominins likely experienced across past social and ecological landscapes as well as within variable contexts of cultural learning.

The increased potential for individuals who live in fission–fusion social systems to transmit cultural information (Romano et al. 2022; van Boekholt et al. 2021) while simultaneously providing individual chimpanzees the opportunity to balance ecological and social pressures (Boesch and Boesch-Achermann 2000; Lehmann and Boesch 2004; Matthews et al. 2021) cannot be overlooked. Fission–fusion social systems foster small-world information transmission clusters that are some of the most efficient configurations documented in primates for transmitting socially learned behaviors and acquiring local cultural variants (van Boekholt et al. 2021). We observed greater social complexity at both the individual and group levels in the termite gathering context compared to the *Ficus* context. This likely supports the idea that choosing to associate with many potential mates in the *Ficus* feeding context likely offers reproductive benefits, whereas termite gathering is a setting where individuals selectively chose to association with others in ways that confers benefits to cultural transmission. Given that the core of our data are records of party co-attendance, these results are likely attributable to the chimpanzees that we studied making decisions to gather termites often, in more selectively constructed parties with vast arrays of different partners in almost every visit compared to *Ficus* feeding parties that are homogeneously composed of similar partners each time. To what extent the patterns of choices that we observed are mediated by cognitive mechanisms, or the timing of other available resources remain to be investigated.

Our results also extend, conceptually, to models of pathogen transmission. Unlike large, cohesive groups where information and pathogens would uniformly spread rather quickly, fission–fusion systems are a balanced solution to reduce the risk of pathogenic transmissibility while facilitating social learning. To produce these benefits, individuals in fission–fusion social systems constantly reshuffle the composition of parties, such that individuals can quickly acquire new information but are not as susceptible to repeated pathogenetic exposures as they would be in longer term associations (Romano et al. 2022). Our use of social niche construction theoretical orientations can be extended to models of cultural information and disease transmission in apes to better understand how context-specific social variation at the individual level might produce differential learning and survival.

While our data do not speak to the characteristics that social niche specialists might possess so as to draw such a diversity of conspecifics to selectively associate with them in some contexts but not others, we anticipate that the unique skills and competencies that these individuals possess are a large contributing factor. Our results of context-specific social niche specializations and social complexity provide preliminary support for the notion that these two variables (particularly, in tool using contexts) likely influence the efficiency of cultural transmission, and potentially even the accumulation of cultural variants. Further, the developmental influence of maternal social niche specialization on the ontogeny of immature individuals is an interesting area of investigation (see Smith et al. 2022). The implications of social niche construction and specialization compounding over many generations via extragenetic inheritance is an exciting theoretical advancement for understanding specific evolutionary mechanisms that promote interindividual and intraspecific variation (Boyd et al. 2011; Lewens 2019). In combination with accounts of functional teaching via tool transfers during sequential tool-use by the Goualougo chimpanzees (Musgrave et al. 2020, 2016; Sanz et al. 2009, 2004, 2010; Sanz and Morgan 2007, 2009, 2011), our results aid in advancing the hypothesis that associating with a wider variety and more diverse set of conspecifics in tool-use contexts might expedite the inter-generational transmission and possible multi-generational accumulation of cultural variants. Furthermore, selective attention toward specific models in the context of social learning has also been documented in human and nonhuman primates (e.g., prestige-bias social learning: Lee and Yamamoto 2023). For example, it has been proposed that novice chimpanzees learn to use stone tools to crack nuts from more knowledgeable or particularly skilled, older models (Boesch et al. 2019; Matsuzawa et al. 2001). Chimpanzees have also been reported to bias their attention toward models who possess higher social status, have more experience, or display greater task proficiency (Kendal et al. 2015; Vale et al. 2014). With growing interest in comparing the mechanisms of cultural diversity across chimpanzee communities (Badihi et al. 2023; Girard-Buttoz et al. 2022; Nakamura and Nishida 2013; van Leeuwen 2021; van Leeuwen et al. 2018) and evidence of stone tool use by australopithecines (Sterelny, 2011), our results can inform predictions regarding the associations between social niche specialists, variable social complexity, and biased social transmission in the cultural contexts of hominids and other animals. In considering the evolution of technology and uniquely hominid characteristics, the conservation of chimpanzee cultures, and the ability to provide chimpanzee-typical social environments in captive settings, it is ever-pressing that we begin to consider the role that goal-oriented social choices and context-specific social complexity play in constructing great ape societies.

Given that the application of wild chimpanzee behavior to benchmark the wellbeing of their captive counterparts is commonplace (Pruetz and McGrew 2001; Veasey et al. 1996), the likely functional, context-specific social niche construction and social complexity that we describe here can also be used to inform the wellbeing of zoo- and sanctuary-living chimpanzees. For example, new models could be generated for monitoring the social wellbeing of species who naturally live in fission–fusion societies that extend beyond considerations of group size and demography (Angley et al. 2024; Neal Webb et al. 2019; Ross 2020; Schel et al. 2013). Equally pressing is the need to elucidate the presence and function of community-specific patterns of social complexity and cultural variation across apes to ensure that this component of ape societies is included in conservation strategies. The conservation of animal cultures is a growing priority (Brakes et al. 2019). The United Nations Environment Program’s Convention on the Conservation of Migratory Species (CMS) of Wild Animals has begun to prioritize the development of policy to identify population-specific repertoires of socially learned knowledge in animal societies and protect their cultures as an integral part of natural biodiversity (CMS 2018). Of additional importance is the potential of specific behavioral and ecological contexts that promote variation in chimpanzee cultural repertoires across populations (Carvalho et al. 2022). Several threats to the conservation of chimpanzees, their habitats, and culture are linked to human disturbance (i.e., habitat loss, degradation, conversion, expansion of roads, mechanized logging, and existing and emerging infectious diseases: Kuhl et al. 2019; Morgan et al. 2018, 2019; Stokes et al. 2010; Strindberg et al. 2018). While also relying on *Ficus* spp. and *Macrotermes* spp. as an important part of their diets, we found these contexts to be important in supporting variation in behavioral diversity and social complexity. Protecting these specific environmental settings of chimpanzee cultural behaviors should be of paramount concern (i.e., cultural heritage sites: Kuhl et al. 2019). For example, certified selective logging activities that occur on a rotational schedule across multiple decades were associated with decreased overall abundance and species diversity of *Ficus* spp. Although not intentionally disturbed while logging, a comparison of neighboring intact forest with selectively logged areas found the timber production forest to contain fewer *Ficus* (Morgan et al. 2019). Further, epigeal and subterranean termite nests are culturally significant contexts for the use of tool sets in the Goualougo Triangle and across the Congo Basin (Deblauwe et al. 2006; McGrew and Rogers 1983; Morgan et al. 2023; Sanz et al. 2004; Suzuki et al. 1995). Like the species and sites of other types of localized chimpanzee cultures, these ecological features require enhanced protection and conservation action (e.g., nut cracking, honey pounding, ant dipping, algae fishing, stone throwing, foraging for crabs: Boesch et al. 2017; Carvalho et al. 2022; Kalan et al. 2019; Koops et al. 2019; Kuhl et al. 2019; Sanz and Morgan 2009; Sanz et al. 2010). Without the necessary safeguards to conserve chimpanzee populations and all the mechanisms that permit the construction of social niches that foster the social transmission of behaviors, unique chimpanzee cultures could diminish or even vanish.

## Supplementary Information

Below is the link to the electronic supplementary material.

Supplementary file1 (PDF 136 KB)

## Data Availability

The datasets supporting this study's findings are available from the authors upon reasonable request.
