# Supplementary material for: Chimpanzees employ context-specific behavioral strategies within fission–fusion societies
Source: Primates. 2024 Oct 19;65(6):541–55. doi: 10.1007/s10329-024-01165-1 (PMC11561109; doi:10.1007/s10329-024-01165-1)
Supplement: Supplementary file 1 — Supplementary file1 (PDF 136 KB) [file 10329_2024_1165_MOESM1_ESM.pdf]

Research article: Primates | *Twenty-five years of primate research in the Ndoki forest*

Chimpanzees employ context-specific behavioral strategies within fission-fusion societies

Jake A. Funkhouser[1,2]*, Stephanie Musgrave[3], David Morgan[4], Severin Ndassoba Kialiema[5], Delon Ngoteni[5], Sean Brogan[5], Philip McElmurray[1], and Crickette Sanz[1,5]

[1] Department of Anthropology, Washington University in Saint Louis, One Brookings Drive, Saint Louis, MO 63130, U.S.A.

[2] Department of Evolutionary Anthropology, University of Zurich, Winterthurerstrasse 190 8057 Zurich, Switzerland

[3] Department of Anthropology, University of Miami, 5202 University Drive, Coral Gables, FL 33146, U.S.A

[4] Fisher Center for the Study and Conservation of Apes, Lincoln Park Zoo, 2001 N. Clark Street, Chicago, IL 60614, U.S.A.

[5] Wildlife Conservation Society, Congo Program, B.P. 14537, Brazzaville, Republic of Congo

*Correspondence: jake.funkhouser@uzh.ch, jakefunkhouser@wustl.edu

SUPPLEMENTARY INFORMATION

TABLE S1: Interlayer correlation results of temporal consistency in social network structure

CONTEXT	$TIME_1$	$TIME_2$	R
Ficus network	2014	2015	0.69
Ficus network	2014	2016	0.54
Ficus network	2014	2017	0.24
Ficus network	2014	2018	0.25
Ficus network	2014	2019	0.16
Ficus network	2015	2016	0.73
Ficus network	2015	2017	0.39
Ficus network	2015	2018	0.39
Ficus network	2015	2019	0.27
Ficus network	2016	2017	0.48
Ficus network	2016	2018	0.49
Ficus network	2016	2019	0.46
Ficus network	2017	2018	0.46
Ficus network	2017	2019	0.40
Ficus network	2018	2019	0.77
Termite gathering	2014	2015	0.85
Termite gathering	2014	2016	0.65
Termite gathering	2014	2017	0.58
Termite gathering	2014	2018	0.43
Termite gathering	2014	2019	0.35
Termite gathering	2015	2016	0.81
Termite gathering	2015	2017	0.65
Termite gathering	2015	2018	0.45
Termite gathering	2015	2019	0.40
Termite gathering	2016	2017	0.72
Termite gathering	2016	2018	0.48
Termite gathering	2016	2019	0.48
Termite gathering	2017	2018	0.73
Termite gathering	2017	2019	0.75
Termite gathering	2018	2019	0.80
Ficus network	2014-2019	mean edge overlap: R = 0.37	
Termite gathering	2014-2019	mean edge overlap: R = 0.39	

<u>*Note*</u>: P-values for all pair-wise comparisons: $P < 0.001$.
